# Apocynin exerts cytoprotective effects on dexamethasone‐induced osteoblasts by inhibiting oxidative stress through the Nrf2 signalling pathway

**DOI:** 10.1111/jcmm.17974

**Published:** 2023-09-25

**Authors:** Xinglong Zhang, Ran Pang, Kai Zhang, Qian Xu, Chunlei Xu, Wei Shi, Xinyu Liang, Dong Li, Wenhao Cui, Shucai Bai, Zhijun Li, Hui Li, Huafeng Zhang

**Affiliations:** ^1^ Department of Orthopaedics TianjinNankai Hospital Tianjin China; ^2^ Department of Orthopaedics General Hospital of Tianjin Medical University Tianjin China; ^3^ School of Integrative Medicine Tianjin University of Traditional Chinese Medicine Tianjin China; ^4^ Department of Pharmacology Kyoto Prefectural University of Medicine Kyoto Japan; ^5^ R&D Center Youjia (Hangzhou) Biomedical Technology Co., Ltd Hangzhou China; ^6^ Department of Orthopaedics Tianjin Hospital Tianjin China

**Keywords:** apocynin, femoral head necrosis, Nrf2 pathway, osteoblast apoptosis, oxidative stress

## Abstract

Steroid‐induced femoral head necrosis (SIFHN) is a serious clinical complication that is caused by prolonged or excessive use of glucocorticoids (GCs). Osteoblast apoptosis and osteogenic differentiation dysfunction caused by GC‐induced oxidative stress and mitochondrial impairment are strongly implicated in SIFHN. Apocynin (APO) is a kind of acetophenone extracted from an herb. In recent years, APO has received much attention for its antiapoptotic and antioxidant properties. This study aimed to investigate whether APO could protect against SIFHN and explore the mechanism. In our study, low‐dose APO had no toxic effects on osteoblasts and restored dexamethasone (Dex)‐treated osteoblasts by improving survival, inhibiting OS and restoring mitochondrial dysfunction. Mechanistically, APO alleviated Dex‐induced osteoblast injury by activating the Nrf2 pathway, and the use of ML385 to block Nrf2 significantly eliminated the protective effect of APO. In addition, APO could reduce the formation of empty lacunae, restore bone mass and promote the expression of Nrf2 in SIFHN rats. In conclusion, APO protects osteoblasts from Dex‐induced oxidative stress and mitochondrial dysfunction through activation of the Nrf2 pathway and may be a beneficial drug for the treatment of SIFHN.

## 1 INTRODUCTION

Long‐term or overuse of glucocorticoids (GCs, i.e. Dexamethasone which is a synthetic glucocorticoid used clinically to treat inflammatory and autoimmune diseases) has been identified as a common risk factor for steroid‐induced femoral head necrosis (SIFHN), which eventually leads to structural damage to the femoral head or even collapse and hip joint destruction.^1^, ^2^ SIFHN progresses due to an imbalance between osteogenesis and osteoclastogenesis.^3^ However, the current method of SIFHN treatment mainly aims to avoid bone resorption, which does not repair bone microarchitecture.^3^ Therefore, protecting osteoblasts might be an effective treatment strategy for SIFHN.

Mounting evidence suggests that oxidative stress (OS) and mitochondrial dysfunction‐mediated apoptosis are strongly connected with SIFHN.^4^, ^5^, ^6^, ^7^ OS represents a disturbance in cellular oxidation and antioxidant effects.^8^ GCs induce the overproduction of reactive oxygen species (ROS) in osteoblasts and inhibit antioxidant enzymes, leading directly to oxidative damage and dysfunction in cells and ultimately to apoptosis.^5^, ^9^ Mitochondria are the primary organelles that generate ROS,^10^ and mitochondrial impairment is a major contributor to apoptosis.^11^ GCs have been reported to induce apoptosis by altering the mitochondrial membrane potential (MMP) of osteoblasts and producing excessive ROS.^11^ Therefore, the key to treating SIFHN is to prevent intracellular OS and restore mitochondrial function in osteoblasts.

Nuclear factor erythroid 2‐related factor 2 (Nrf2) maintains cellular redox homeostasis by promoting the expression of downstream haem oxygenase‐1 (HO‐1) and antioxidant enzymes and plays an important protective role by reducing OS damage and inhibiting apoptosis.^12^, ^13^ The Nrf2 pathway also protects mitochondrial function through the restoration of MMP and reducing mitochondrial ROS production.^14^, ^15^ It has been demonstrated that the Nrf2 pathway is significantly downregulated in GC‐treated osteoblasts, and its activation reduces ROS production, restores mitochondrial function and inhibits apoptosis in osteoblasts.^16^ Thus, this pathway might be a promising therapeutic target for SIFHN.

Recently, natural compounds have shown great promise in the treatment of SIFHN due to their powerful antioxidant properties. Apocynin (APO) is a naturally occurring acetophenone that has been shown to alleviate various diseases such as diabetes, stroke, hypertension and acute pancreatitis.^17^ It has been demonstrated that APO activates the Nrf2 signalling pathway to exert its antioxidant effects.^18^, ^19^, ^20^ However, it is not yet clear whether APO is protects against SIFHN. Consequently, the objective of the current study was to study apoptosis and OS physiology and its signalling system and identify the effects of APO on dexamethasone (Dex)‐induced apoptosis in osteoblasts.

## 2 MATERIALS AND METHODS

### 2.1 Reagents

Apocynin (purity ≥99.96%), dexamethasone (purity ≥97%) and ML385 were acquired from MedChemExpress (MCE). The Cell Counting Kit‐8 (CCK‐8) kit, Annexin‐V/7‐AAD Apoptosis Detection Kit, DCFH‐DA Detection Kit, Mitochondrial Superoxide (Mito‐Sox) kit and JC‐1 Kit were obtained from Beijing Solarbio Science & Technology (Beijing, China). Primary antibodies against 4‐hydroxynonenal (4‐HNE, ab48506), 8‐hydroxy‐2′‐deoxyguanosine (8‐OhdG, ab62623), cleaved caspase 3 (C‐caspase3, ab2302), BAX (ab3191), BCL‐2 (ab241548), alkaline phosphatase (ALP, ab229126), osteoprotegerin (OPG, ab73400), RUNX2 (ab76956), Nrf2 (ab137550) and HO‐1 (ab302671) were supplied by Abcam (Cambridge, MA, USA).

### 2.2 Cell culture and drug treatments

MC3T3‐E1 cells were purchased from Procell (Wuhan, China) and maintained in α‐modified essential medium (α‐MEM) supplemented with 10% fetal bovine serum (FBS, Gibco) and 1% penicillin–streptomycin (Gibco) at 37°C in a 5% CO_2_ environment. To establish the SIFHN cell model, osteoblasts were cultured in medium containing 1 μM dexamethasone for 24 h. For APO treatment, osteoblasts were exposed to 1, 10 and 100 μM APO for 24 h. In the Dex + APO group, osteoblasts were pretreated with diverse concentrations of APO (1, 10, 100 μM) for 2 h and then conditioned with Dex for 24 h. In the Dex + APO + ML385 group, osteoblasts were pretreated with ML385 (5 μM) for 24 h, followed by APO (100 μM) treatment for 2 h, and then stimulation with Dex for 24 h.

### 2.3 Proliferation assay

The CCK‐8 kit was used to investigate the cytoprotective effects of APO on osteoblasts. When the cells reached 80% confluence, the osteoblasts were treated with different concentrations of APO (0.01, 0.1, 1, 10, 100, 1000 μM) for 24 h. To explore the cytoprotective effect of APO on osteoblasts, MC3T3‐E1 cells were pretreated with various concentrations of APO (1, 10 and 100 μM) for 2 h with or without 1 μM Dex treatment. Subsequently, CCK‐8 (10 μL) working solution was added dropwise to each well and incubated for 4 h. Finally, the optical density (OD, 450 nm) was recorded with a microplate reader.

### 2.4 Flow cytometry

Flow cytometry was used to estimate the apoptosis rate and reactive oxygen levels in osteoblasts in each group. The cells were harvested after centrifugation and resuspension. Subsequently, the cells were stained with 5 μL of Annexin‐V and 7‐AAD solution and incubated for 20 min at room temperature. Then, we performed flow cytometry (BD Biosciences) to detect apoptosis. To detect intracellular ROS levels, different interventions were implemented when the cells reached 80% confluence. Osteoblasts were labelled with DCFH‐DA and incubated for 20 min at 37°C. Then, ROS levels were detected by flow cytometry (BD Biosciences, USA).

### 2.5 Mitochondrial function assay

The MMP and the levels of mitochondrial superoxide (Mito‐Sox) in each group of MC3T3‐E1 cells were measured using a JC‐1 Assay Kit and Mito‐Sox Red fluorescence assay probe.

### 2.6 Western blotting

Total protein was extracted from osteoblasts by RIPA lysis buffer. The protein concentration was determined by a BCA reagent, and then equal amounts of samples were added to each lane. The proteins were separated in 10%–15% SDS–PAGE gels and then electrotransferred to polyvinylidene fluoride (PVDF; Millipore, MA, USA) membranes. Subsequently, the membraned were blocked in 5% skim milk for 1 h and then incubated with antibodies against BAX (1:1000), BCL‐2 (1:2000), ALP (1:2000), OPG (1:1000), RUNX2 (1:1000), Nrf2 (1:1000), HO‐1 (1:1000) and β‐actin (1:1000) overnight at 4°C. After the blot was incubated with secondary antibodies (1:5000) for 1 h, the specific bands were detected and quantitatively analysed by a chemiluminescence system.

### 2.7 Detection of osteoid differentiation and mineralization

The activity of alkaline phosphatase (ALP) was determined to evaluate whether APO could reduce Dex‐mediated osteoblast damage. Cells (5 × 104 each well) were inoculated in 12‐well plates and supplemented with osteogenic induction medium after the different drug interventions. After 1 week, the cells were lysed with 100 μL of working solution, and ALP reagent kits (Beyotime, China) were used to measure ALP activity.

Alizarin Red S staining was employed to detect calcium deposition in osteoblasts by complexing calcium ions with Alizarin Red S. After osteogenic induction in the different treatment groups, the cells were stained with ARS on Day 21 (Cyagen, China).

### 2.8 Drug targets identified by bioinformatics analysis

To further explore the mechanism by which APO attenuates OS in osteonecrosis of the femoral head, we used bioinformatics tools to search for therapeutic targets of APO. First, genes associated with femoral head necrosis were collected from the CTD database (https://ctdbase.org/), and then the Amigo2 database (http://geneontology.org/) was searched for genes involved in OS using the words ‘oxidative stress’. Finally, we searched for potential targets of APO in the SuperPRED database (https://prediction.charite.de/). The intersection was determined by constructing Venn diagrams and was used to identify potential targets by which APO regulates OS in femoral head necrosis. To understand the biological process (BP), cell component (CC), molecular function (MF) and pathways in which these targets were involved, enrichment analysis of these targets was performed using the R package clusterProfiler with the minimum gene set to 5 and maximum gene set to 5000. A *p*‐value of <0.05 and a false discovery rate (FDR) of <0.1 were considered statistically significant. Protein–protein interaction (PPI) network maps of the targets were built from the STRING database (https://cn.string‐db.org/). The results were imported into Cytoscape software, and the significant nodes in the PPI were calculated using the BottleNeck algorithm of the cytoHubba plugin.

### 2.9 Immunofluorescence staining

After the various treatments, the cells were fixed and permeabilized in a humidified chamber for 15 min. We then blocked the cells with 3% BSA and incubated them overnight with anti‐Nrf2 (dilution 1:150), HO‐1 (dilution 1:200), 4‐HNE (dilution 1:200), 8‐OHdG (dilution 1:200) and cleaved caspase 3 (dilution 1:200) primary antibodies and then incubated then with rabbit anti‐rat secondary antibodies for 2 h in the dark. The mean fluorescence intensity (MFI) was analysed using a fluorescence microscope.

### 2.10 Animal model and groupings

Twenty‐four 12‐week‐old Sprague–Dawley rats (male, 380 ± 50 g) were obtained from the Tianjin Medical University Experimental Animal Center and maintained under specific pathogen‐free (SPF) conditions at 23°C. According to the experimental requirements, after 1 week of adaptation, the animals were grouped as follows: negative control (NC) group, Dex treatment group (Dex) and APO pretreatment group (Dex + APO), *n* = 8/per group). The Dex group received daily intramuscular injections of 21 mg/kg Dex for 28 days following to the previous protocol.^21^ The Dex + APO group received 100 mg/kg APO daily by gavage 1 h before Dex treatment. For the NC group, rats were administered normal saline daily.

Four weeks later, the rats were sacrificed under isoflurane anaesthesia, and the femurs were extracted and preserved in 4% paraformaldehyde for 48 h.

### 2.11 MRI

MRI was performed on bilateral femurs using a 9.3 T small‐animal scanner (Bruker, Germany) after isoflurane euthanasia, and image analysis was conducted using ImageJ software as previously described.^22^ The image signal intensity of the femoral head was averaged over the region of interest, which was manually drawn on the original image and normalized to the average image intensity of adjacent muscle regions calculated over the same size region of interest.

### 2.12 Micro‐CT analysis

Micro‐CT (Bruker, Aartselaar, Belgium) was used to assess the microstructure of the rat femoral head. The following parameters were analysed using CT Analyzer software (Bruker): the bone volume fraction (BV/TV, %), trabecular thickness (Tb.Th, mm), mean trabecular number (Tb.N, 1/mm), trabecular spacing (Tb.Sp, mm) and bone mineral density (BMD, g/cm^2^).

### 2.13 Histology and immunofluorescence staining

First, the fixed femoral head was decalcified with EDTA for 6 weeks, followed by ethanol gradient dehydration and xylene transparency. Subsequently, paraffin embedding was performed, and the femoral head was cut into 5‐μm‐thick sections. Routine HE staining was executed to observe the pathological changes in subchondral bone within the femoral head. For immunofluorescence staining, after deparaffinization and rehydration, the sections were incubated with primary antibodies against Nrf2, HO‐1,4‐HNE, cleaved caspase 3 and 8‐OHdG overnight and then incubated with rabbit anti‐rat secondary antibodies for 120 min in the dark. Five randomly selected fields per section and three sections per rat were analysed to determine the percentage of positive cells per field.

### 2.14 TUNEL assay

Cell and tissue apoptosis were detected by a TUNEL Assay Kit (Beyotime, China). To examine apoptosis, 1 × 104 cells were seeded on coverslips for 24 h before the various treatments. Then, the cells were subjected to fixation and permeabilization. After dewaxing and rehydration, the paraffin sections were incubated in TUNEL working solution, and 4′,6‐diamidino‐2‐phenylindole (DAPI) was added to counterstain the cell nuclei. Images were acquired using a fluorescence microscope.

### 2.15 GSH, CAT, GPx and SOD assays

OS levels in serum and MC3T3‐E1 cells were detected by reduced glutathione (GSH), catalase (CAT), glutathione peroxidase (GPx) and superoxide dismutase (SOD) assay kits (KeyGEN BioTECH, China).

### 2.16 Ethics statement

Tianjin Medical University General Hospital's Animal Research Ethics Committee authorized all animal experimentation protocols in accordance with the US National Institutes of Health's guidelines for the care and use of animals.

### 2.17 Statistical analysis

All experiments were repeated at least three times. The data are expressed as the mean ± standard deviation (SD). Comparisons between the two groups were assessed by Student's *t*‐test for mean differences or one‐way analysis of variance with a Bonferroni/Dunn post hoc test for multiple comparisons. Statistical analysis was conducted using GraphPad Prism 8.0.2 (United States) (*p < 0.05, **p < 0.01, ***p < 0.001).

## 3 RESULTS

### 3.1 APO alleviated Dex‐induced injury in MC3T3‐E1 cells

**FIGURE 1:**
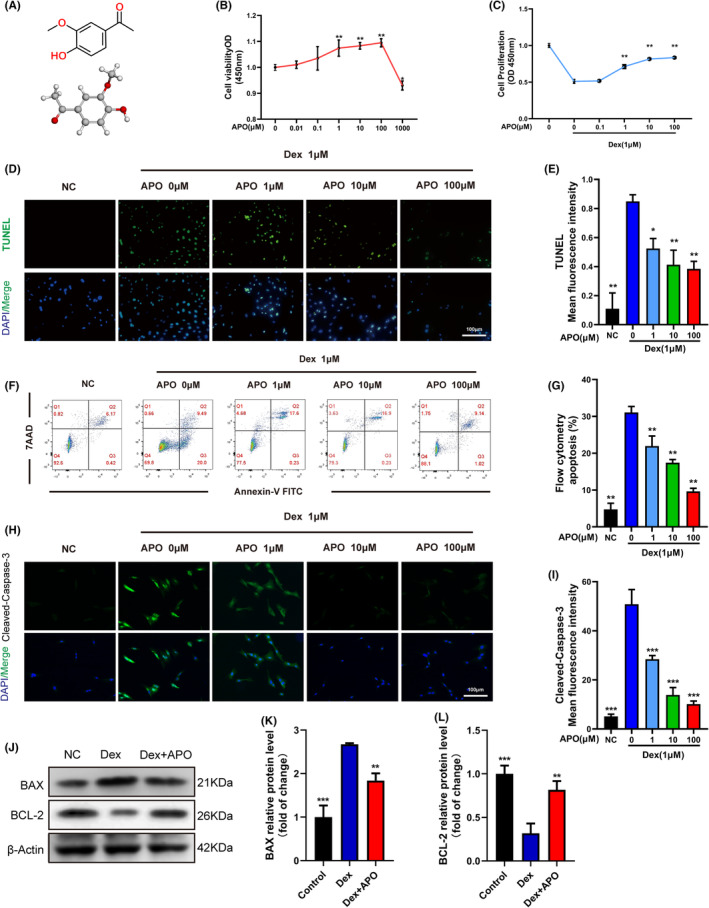
Cytoprotective effect of APO pretreatment on the viability of Dex‐induced osteoblasts. (A) The chemical structure of APO. (B) Osteoblasts were incubated with various concentrations of APO for 24 h, and viability was examined with CCK‐8 assays. (C) Osteoblasts were pretreated with the indicated concentration of APO for 2 h and then exposed to Dex for 24 h. Then, viability was analysed by CCK‐8 assays. (D–G) Osteoblast apoptosis was analysed by TUNEL staining and flow cytometry. (H, I) Quantitative analysis of the MFI after C‐caspase3 staining. (J–L) Western blot of the expression of BAX and BCL‐2. Scale bar, 100 μm.

The molecular formula of APO is HOC_6_H_3_(OCH_3_)COCH_3_, and its chemical structure is illustrated in Figure 1A. Firstly, to investigate the toxicity of APO on osteoblasts, we treated osteoblasts with different concentrations of APO for 24 h. The CCK‐8 results showed that 100 μM was the maximum safe concentration (Figure 1B). Therefore, we selected APO concentrations of 1, 10 and 100 μM for subsequent experiments. In addition, Dex (1 μM) significantly reduced osteoblast activity, and APO mitigated this effect of Dex on osteoblasts (Figure 1C). The TUNEL staining (Figure 1D,E) and flow cytometry (Figure 1F,G) results showed that Dex significantly increased the osteoblast apoptosis ratio, and APO exerted a significant cytoprotective effect. To investigate whether the mitochondrial pathway was responsible for the antiapoptotic effect of APO, western blotting and immunofluorescent staining were performed to measure the expression of proteins related to the mitochondrial pathway. The expression of BAX and the mean fluorescence intensity of C‐caspase3 were increased significantly, while BCL‐2 protein expression was considerably decreased after Dex treatment (Figure 1H–L). APO pretreatment dramatically prevented this change in the experimental group. In conclusion, APO significantly increased cell survival and inhibited apoptosis in osteoblasts induced by Dex.

### 3.2 APO attenuated Dex‐induced osteogenic differentiation dysfunction in MC3T3‐E1 osteoblasts

**FIGURE 2:**
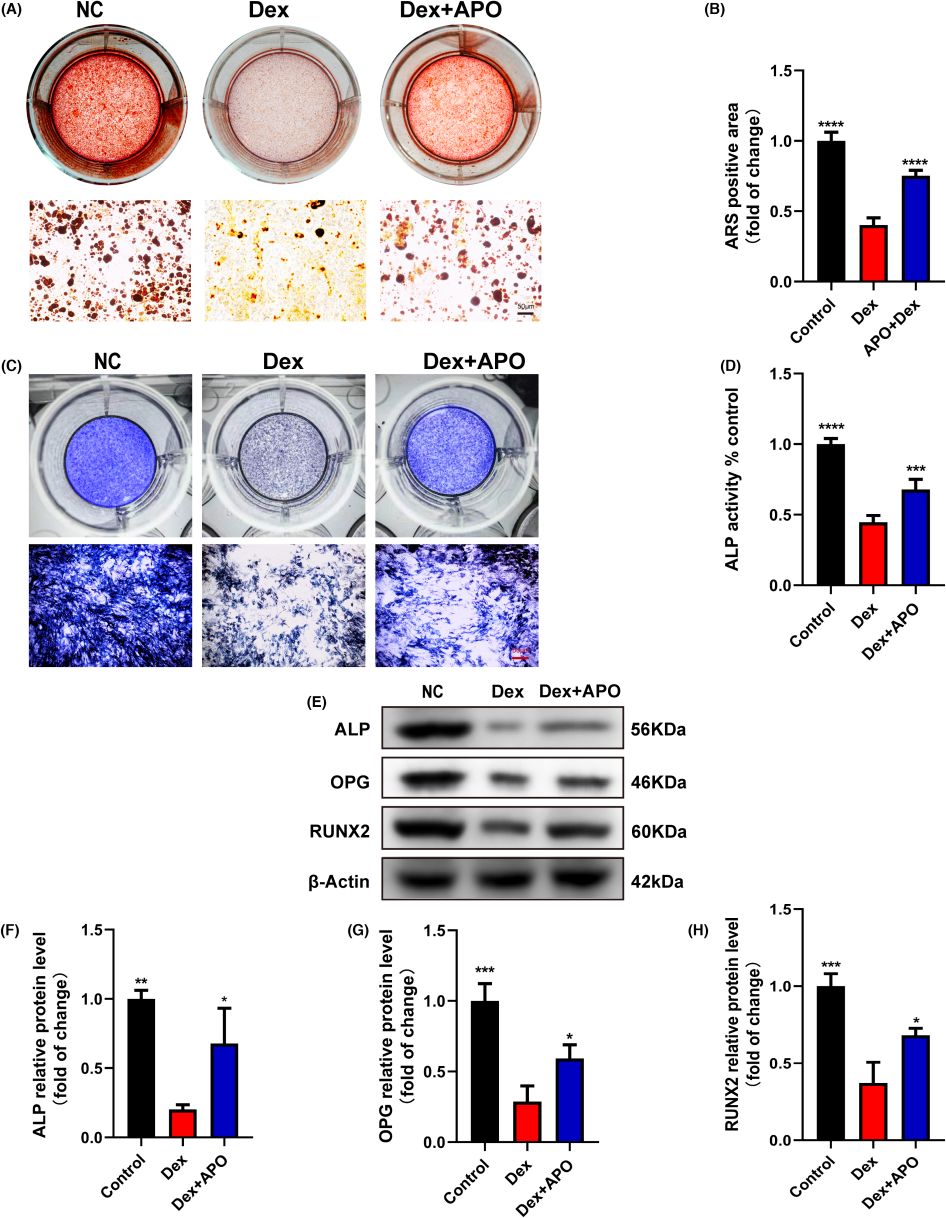
APO restored osteogenic differentiation and mineralization in osteoblasts induced by Dex. After APO (100 μM) pretreatment for 2 h, osteoblasts were exposed to Dex (1 μM) for 24 h in the Dex + APO group. (A) ARS staining measured the mineralization potential of osteoblasts after 21 days of osteogenic induction. (B) Quantitative analysis of the ARS‐positive area. (C, D) ALP activity in osteoblasts was examined after 7 days of osteogenic induction. (E–H) Western blot of the expression of ALP, OPG and RUNX2. Scale bar, 50 μm.

The osteogenic differentiation and mineralization abilities of osteoblasts were examined. Figure 2A,B shows that Dex treatment significantly decreased the number of calcified nodules in osteoblasts, while APO pretreatment significantly restored the mineralization capacity of osteoblasts compared with that in the Dex group. In addition, Dex significantly suppressed ALP activity in osteoblasts, while APO pretreatment markedly enhanced ALP activity (Figure 2C,D). We also discovered Dex downregulated the levels of ALP, OPG and RUNX2, which indicated that Dex impaired osteogenic differentiation (Figure 2E–H). However, APO pretreatment upregulated the protein expression of ALP, OPG and RUNX2. These findings suggested that Dex inhibited the osteogenic differentiation and mineralization potential of osteoblasts, and APO ameliorated osteogenic differentiation dysfunction.

### 3.3 APO protected osteoblasts by inhibiting the production of ROS

**FIGURE 3:**
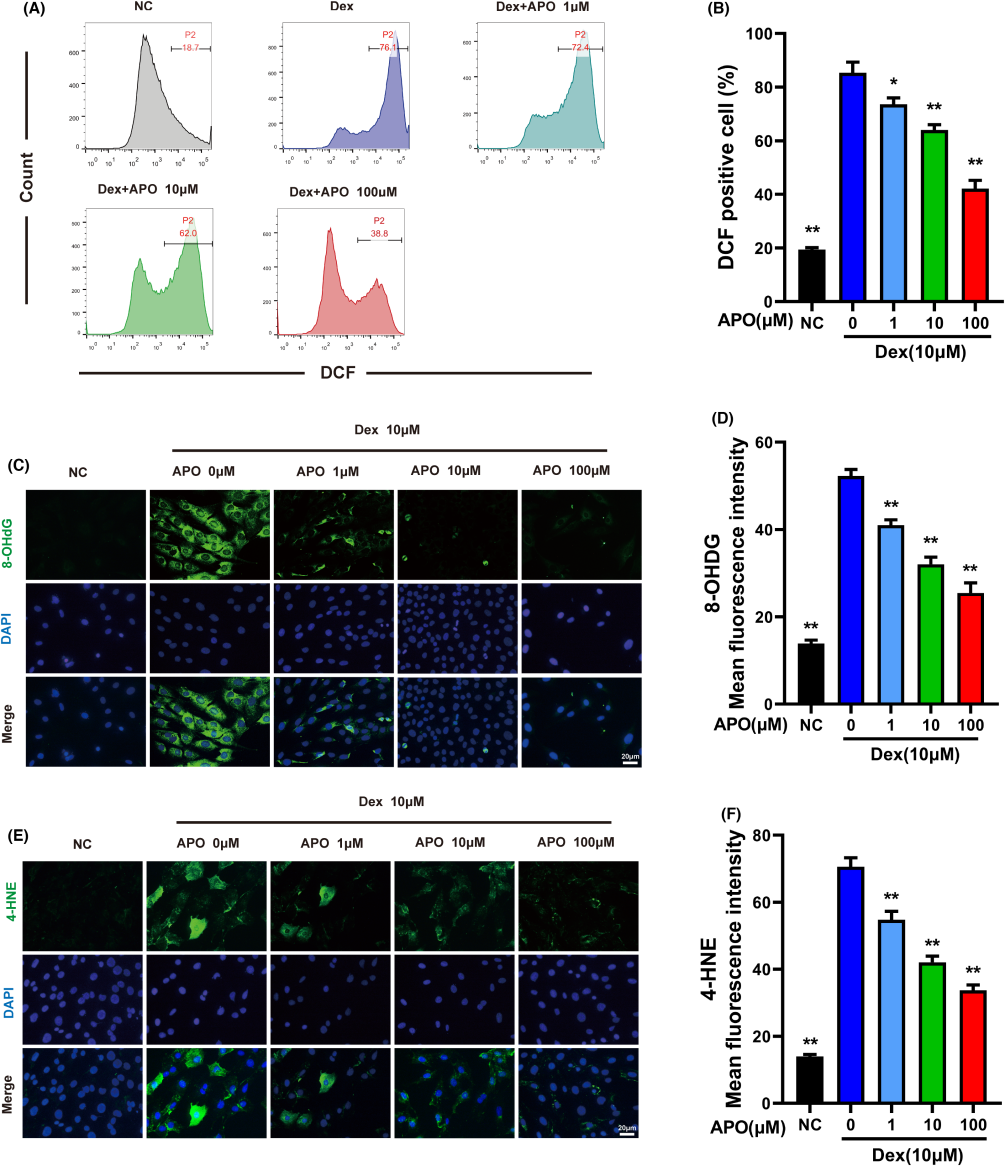
APO inhibited Dex‐induced ROS generation in MC3T3‐E1 cells. (A) Intracellular ROS levels in osteoblasts were determined by flow cytometry. (B) Quantitative analysis of the ROS levels in the different groups. (C) Representative 8‐OHdG immunofluorescence staining image. (D) Quantitative analysis of the MFI after 8‐OHdG staining. (E) Representative 4‐HNE immunofluorescence staining image. (F) The MFI of 4‐HNE immunofluorescence staining was analysed. Scale bar, 20 μm.

Many studies have revealed that ROS are critical in the proapoptotic signalling cascade^23^, ^24^; therefore, we used flow cytometry and immunofluorescence staining to explore the effect of APO on ROS generation in MC3T3‐E1 cells. Our results demonstrated that Dex promoted the formation of ROS compared with those in the NC group, and APO significantly suppressed ROS generation (Figure 3A,B). 4‐HNE and 8‐OHdG are the end products of OS, and the intracellular levels of these products can be used to assess the state of OS. As shown in Figure 3C–F, Dex promoted the intracellular levels of 4‐HNE and 8‐OHdG compared with those in the NC group. However, APO pretreatment significantly inhibited the production of these OS products. OS is an impairment of redox homeostasis caused by excessive ROS and decreased antioxidant enzyme activity in an organism. APO inhibited Dex‐induced ROS production and reduced OS damage in osteoblasts.

### 3.4 APO relieved Dex‐induced mitochondrial dysfunction in osteoblasts in vitro

**FIGURE 4:**
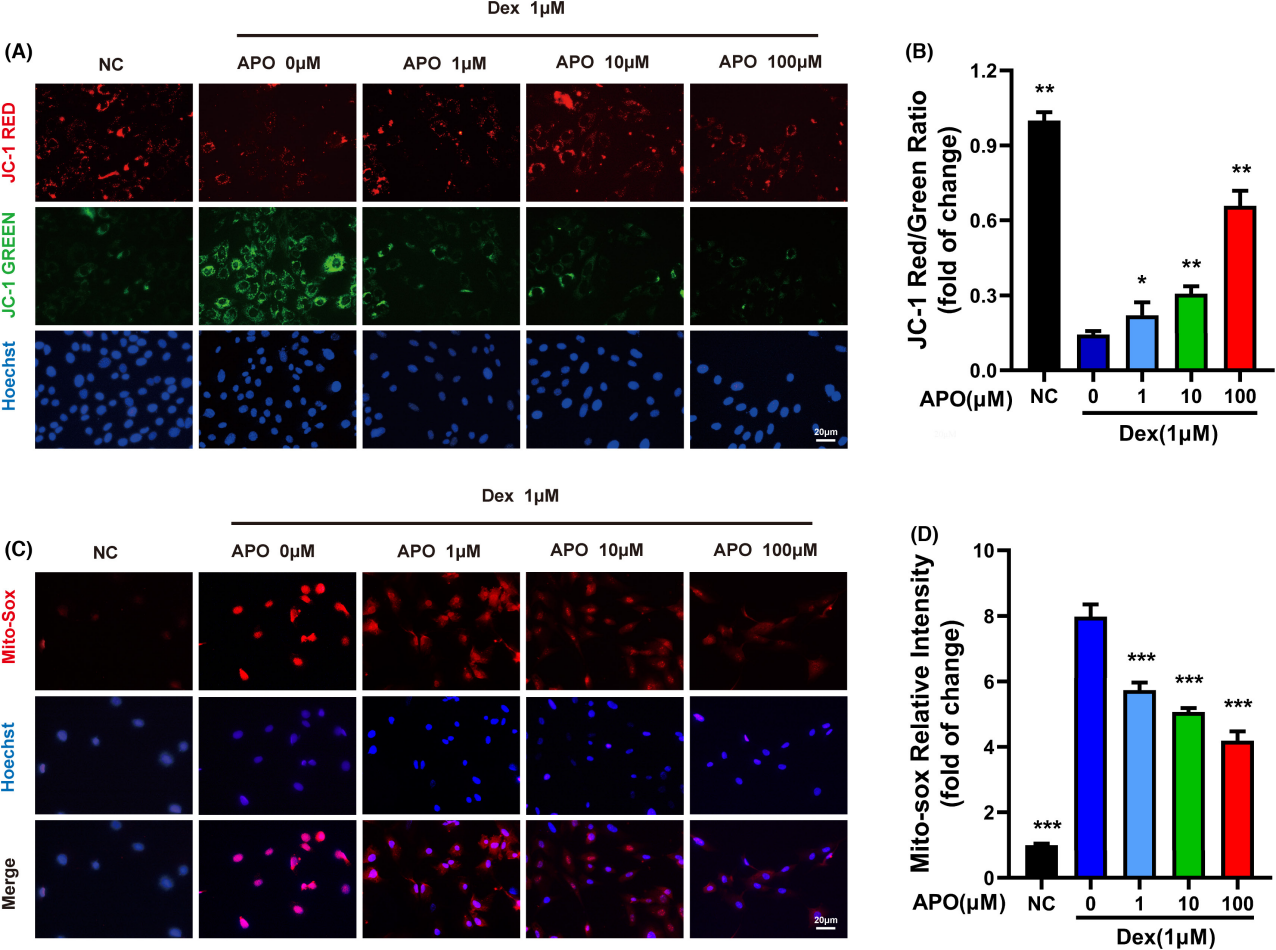
APO restored mitochondrial function in Dex‐treated osteoblasts. Osteoblasts were incubated with 1 μM Dex with or without APO (1, 10, and 100 μM) for 24 h. (A) Representative images of the MMP after staining with JC‐1. Red indicates normal mitochondria, and green indicates MMP depolarization. (B) Fluorescence intensities (ratio of red/green JC‐1 fluorescence) of MC3T3‐E1 cells. (C) Representative images of mitochondrial ROS levels after staining with Mito‐Sox red. (D) MFI of Mito‐Sox red fluorescence. Scale bar, 20 μm.

Mitochondria are the main sites of ROS production, and mitochondrial dysfunction is a major cause of apoptosis. Therefore, we examined the effects of APO on MMP and mitochondrial ROS in Dex‐treated osteoblasts using the probes JC‐1 (Figure 4A) and Mito‐Sox (Figure 4C), respectively. MMP was assessed by determining the ratio between red (aggregated JC‐1) and green (monomeric JC‐1) fluorescence. Compared with control cells, Dex‐treated osteoblasts had a significantly lower MMP and higher levels of ROS, and APO significantly restored the MMP and decreased mitochondrial superoxide anion levels in osteoblasts (Figure 4B,D). In summary, APO restored the MMP and inhibited mitochondrial ROS generation in osteoblasts.

### 3.5 Nrf2 might be a therapeutic target if APO according to bioinformatics analysis

**FIGURE 5:**
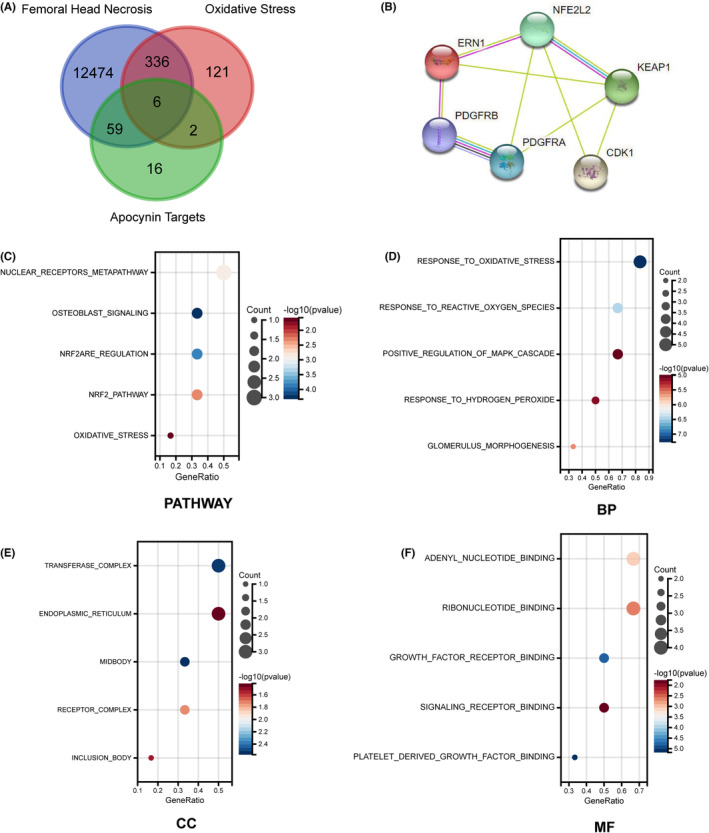
Analysis of potential therapeutic targets of APO, as determined by network pharmacology. (A) Venn diagram showing APO target prediction of OS in osteonecrosis of the femoral head (ONFH). (B) PPI network of 6 targets. (C) Pathways of the potential targets. (D–F) GO enrichment analysis of the targets. Biological process (D), cell component (E) and molecular function (F). BP, biological process; CC, cell component; GO, Gene Ontology; MF, molecular function; PPI, protein–protein interaction.

To further explore the mechanism by which APO attenuates OS in osteoblasts, we identified therapeutic targets of APO by using bioinformatics tools. The Venn diagrams showed six intersecting genes related to osteonecrosis of the femoral head, OS and APO targets: NFE2L2 (Nrf2), ERN1, PDGFRB, PDGFRA, KEAP1 and CDK1 (Figure 5A). PPI network analysis showed that Nrf2 was the most important target (Figure 5B). These genes are involved in a variety of signalling pathways, including the Nrf2 pathway, OS pathway and osteoblast pathway (Figure 5C). Gene annotations and classifications based on BP, CC and MF are shown in Figure 5D–F.

### 3.6 Nrf2 activation was involved in the osteoprotective effect of APO

**FIGURE 6:**
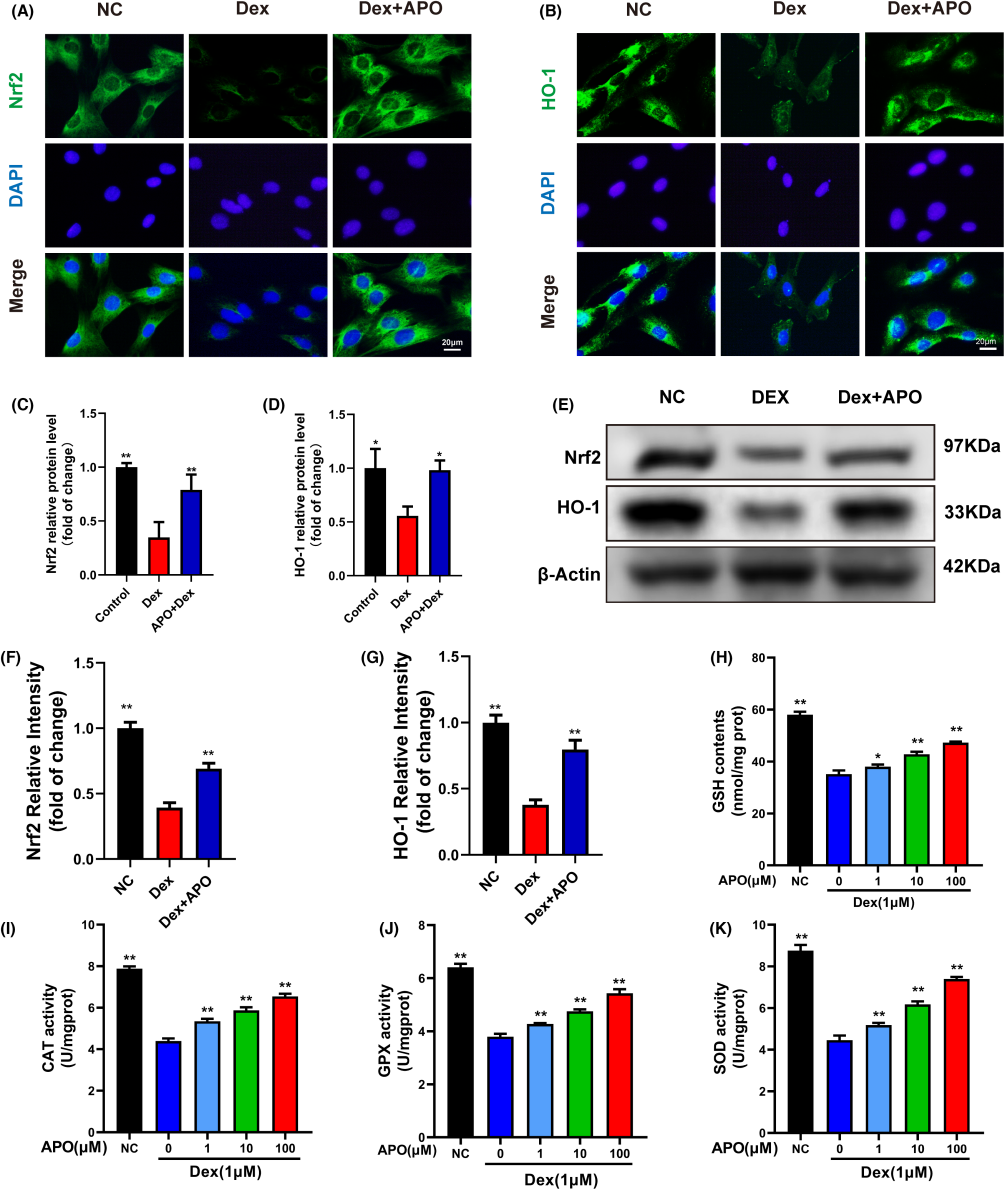
APO activated the Nrf2 pathway and upregulated the expression of antioxidant enzymes. (A, B) Immunofluorescence was conducted to examine the levels of Nrf2 and HO‐1 after 24 h of APO treatment (100 μM). (C, D) The MFI of Nrf2 and HO‐1 in the three groups. (E–G) Western blotting was performed to measure Nrf2 and HO‐1 expression. (H–K) The levels of GSH and the activities of CAT, GPx and SOD. Scale bar, 20 μm. CAT, catalase; GPx, glutathione peroxidase; GSH, glutathione; MDA, malondialdehyde; SOD, superoxidase dismutase.

Nrf2 plays a critical role in cellular sensing of ROS and antioxidant defence.^25^ To further confirm whether the antioxidant effect of APO involved Nrf2 signalling pathway activation, we measured the protein levels of Nrf2, HO‐1 and downstream antioxidant enzymes in vitro. As shown in Figure 6A–D, Dex prominently reduced the fluorescence intensities of Nrf2 and HO‐1, which indicated that the expression of Nrf2 and HO‐1 was inhibited. Consistent with the immunofluorescence staining results, western blot analysis of Nrf2 and HO‐1 showed that Dex markedly downregulated Nrf2 and HO‐1 protein expression, while APO dramatically upregulated their expression (Figure 6E–G). Moreover, as shown in Figure 6H–K, APO pretreatment increased the activity of downstream antioxidant enzymes (GSH, CAT, GPX, SOD) by activating the Nrf2 pathway. These data suggested that the cytoprotective effect of APO antagonized OS damage and cell apoptosis by activating the Nrf2 pathway.

### 3.7 Nrf2 activation improved cell viability and osteogenic differentiation by inhibiting OS and restoring mitochondrial function

**FIGURE 7:**
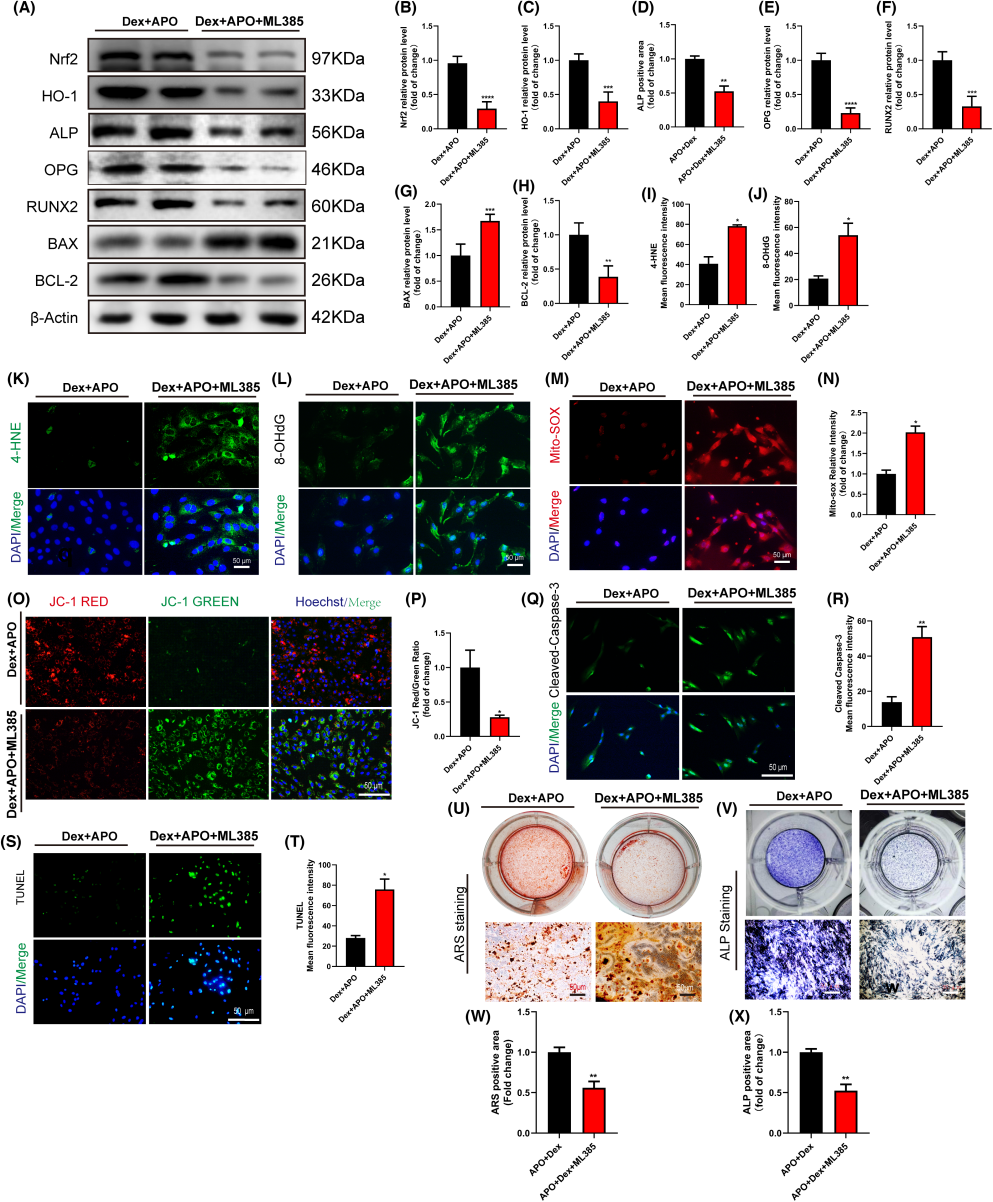
Blocking the Nrf2 pathway neutralized the cytoprotective effect of APO on Dex‐induced osteoblasts. (A–G) Western blotting was used to detect the protein expression of Nrf2, HO‐1, ALP, OPG, RUNX2, BAX and BCL‐2. (I–L) 4‐HNE and 8‐OHdG immunofluorescence staining was used to detect OS levels in MC3T3‐E1 cells. (M–P) Mito‐Sox and JC‐1 staining were performed to assess ROS production and the membrane potential polarization status in mitochondria. (Q–T) C‐caspase3 and TUNEL staining were used to measure apoptosis in MC3T3‐E1 cells. (U–X) ALP activity assays and ARS staining were used to evaluate the osteogenic differentiation of osteoblasts. Scale bar, 50 μm.

We used ML385 (an inhibitor of Nrf2) to block the Nrf2 pathway to examine the role of Nrf2 in the protective effect of APO against oxidative stress. Compared with those in the Dex + APO group, the levels of Nrf2 and HO‐1 were significantly inhibited in the Dex + APO + ML385 group after ML385 (5 μM) treatment (Figure 7A,D–H). In the Dex + APO + ML385 group, blocking the Nrf2 pathway downregulated the expression of osteogenesis‐related markers (ALP, OPG, RUNX2) and the apoptosis‐related marker BCL‐2 and upregulated the expression of BAX (Figure 7A–C). In addition, ML385 treatment impaired the ability of APO to antagonize OS damage and protected mitochondrial function. Therefore, the expression of the oxidative stress‐related markers 4‐HNE and 8‐OHdG (Figure 7I–L) was significantly increased, and mitochondrial function (Figure 7M–P) was notably impaired. As shown in Figure 7Q–T, blocking the Nrf2 pathway markedly increased the fluorescence intensities of TUNEL and C‐caspase3, which suggested that ML385 increased apoptosis in the Dex + APO + ML385 group. ML385 also exerted an inhibitory effect on ALP activity and mineralization in osteoblasts induced by Dex (Figure 7U–X).

### 3.8 APO pretreatment alleviated disease progression in the SIFHN rat model

**FIGURE 8:**
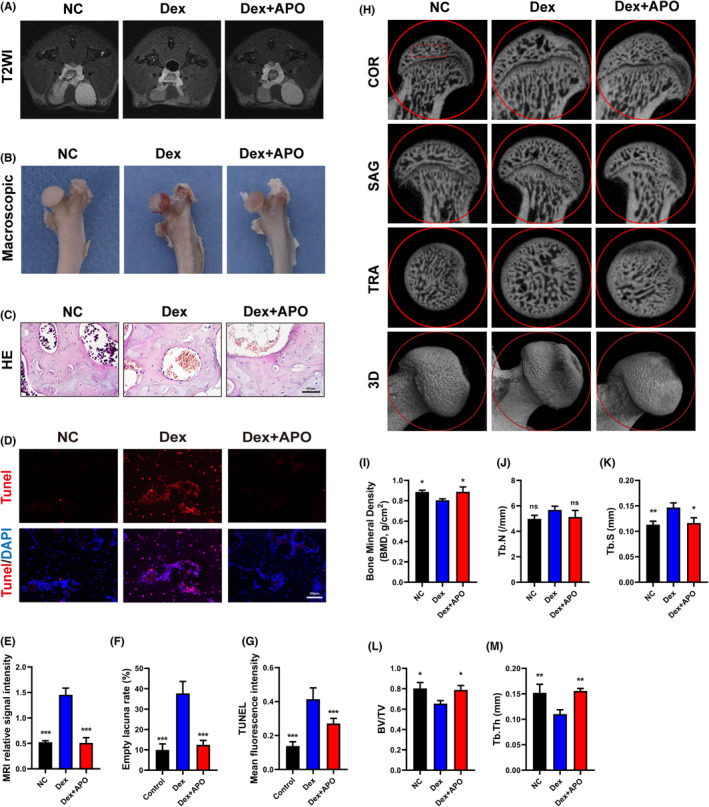
APO relieved the progression of SIFHN. (A, E) Representative MRI images of the rat femoral head (T2WI) and relative MRI signal intensity. (B) Representative macroscopic images of the femoral head in rats. (C, F) Representative images of HE staining. (D, G) Representative image of TUNEL staining and the MFI of TUNEL staining. (H–M) Representative images of micro‐CT scanning and quantitative analysis of BMD (I), Tb.N (J), Tb. S (K), BV/TV (L) and Tb.Th (M). The red rectangle indicates the region of interest (ROI). n.s., not significant. Scale bar, 100 μm.

To investigate the therapeutic effects of APO, we established a rat model of SIFHN. After 4 weeks of treatment, we used MRI, histological and micro‐CT evaluations to assess the protective effect of APO on bone. The T2‐weighted MRI (T2WI) results showed that the femoral head oedema signal was markedly higher in Dex‐treated rats than in the NC group, and the rats in the Dex + APO group had significantly reduced oedema signals in the femoral head (Figure 8A,E). In the NC group, the surface of the femoral head appeared porcelain white with a smooth surface, whereas the articular cartilage surface appeared focally congested and oedematous with the cartilage exhibiting a greyish colour in the Dex group. APO treatment significantly suppressed the congestion and oedema in cartilage and exhibited a colour between that in the NC group and Dex group (Figure 8B). HE staining indicated that empty lacunae and necrotic haematopoietic cells in the subchondral region were apparently more abundant in the Dex group than that in the NC group (Figure 8C,F). In addition, TUNEL staining demonstrated that pretreatment with APO significantly reduced the MFI in the femoral head compared with that in the Dex group (Figure 8D). Micro‐CT revealed that compared to the NC group, Dex significantly damaged the subchondral bone and resulted in marked declines in BV/TV, Tb.Th, Tb. S and BMD. However, APO significantly reversed the toxic effects of Dex on bone reconstruction (Figure 8H–M).

### 3.9 APO restored the activity of antioxidant enzymes and decreased oxidative stress products by upregulating Nrf2 in a rat model of SIFHN

**FIGURE 9:**
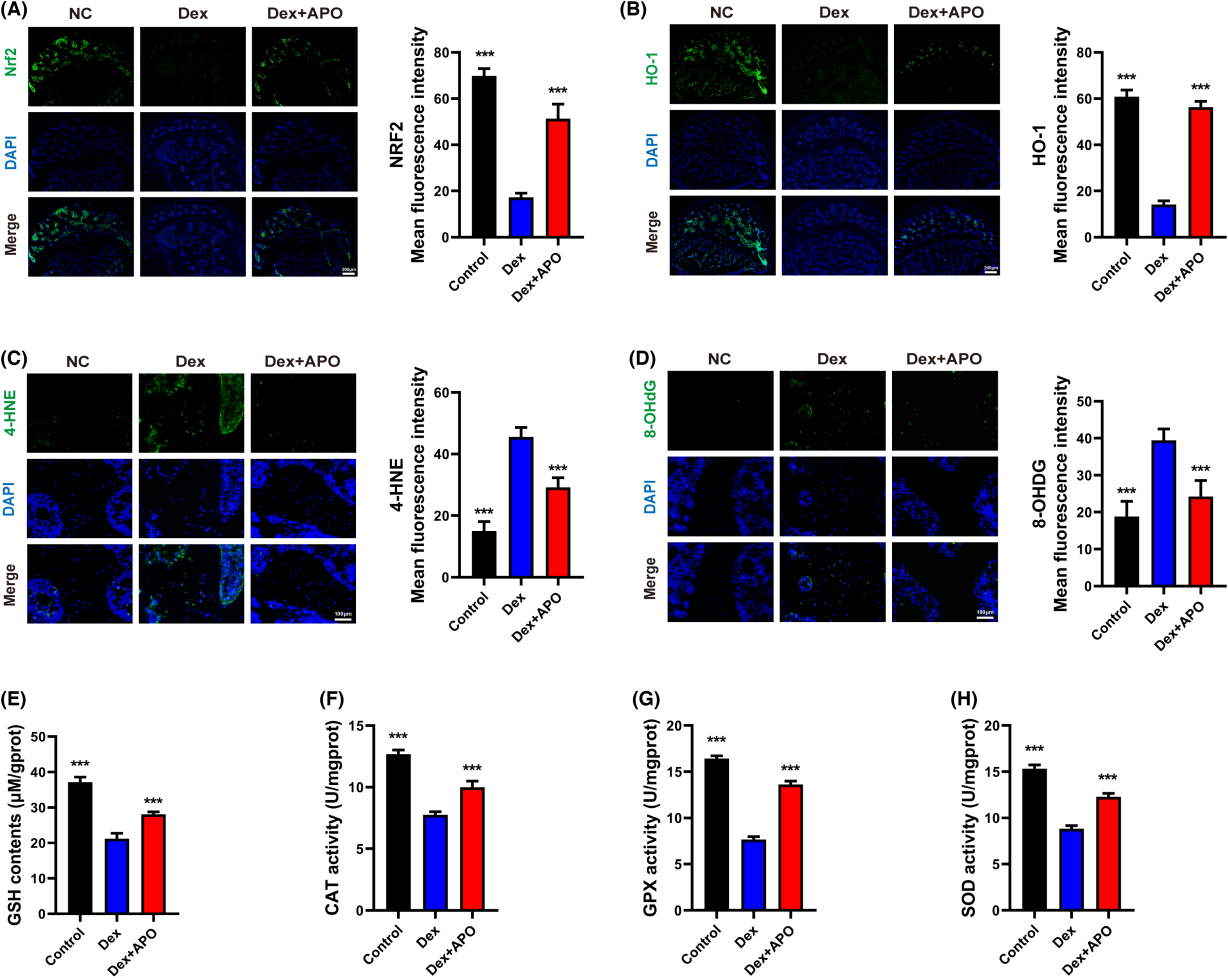
APO promoted Nrf2 expression and suppressed oxidative stress levels in the femoral head. Representative immunofluorescence pictures and quantitative analysis of the MFI of Nrf2 (A), HO‐1 (B), 4‐HNE (C) and 8‐OHdG (D) in the different treatment groups. (E–H) The contents of antioxidant enzymes (GSH, CAT, GPx, SOD) in rat plasma. Scale bar (A, B) = 200 μm, scale bar (C, D) = 100 μm.

**FIGURE 10:**
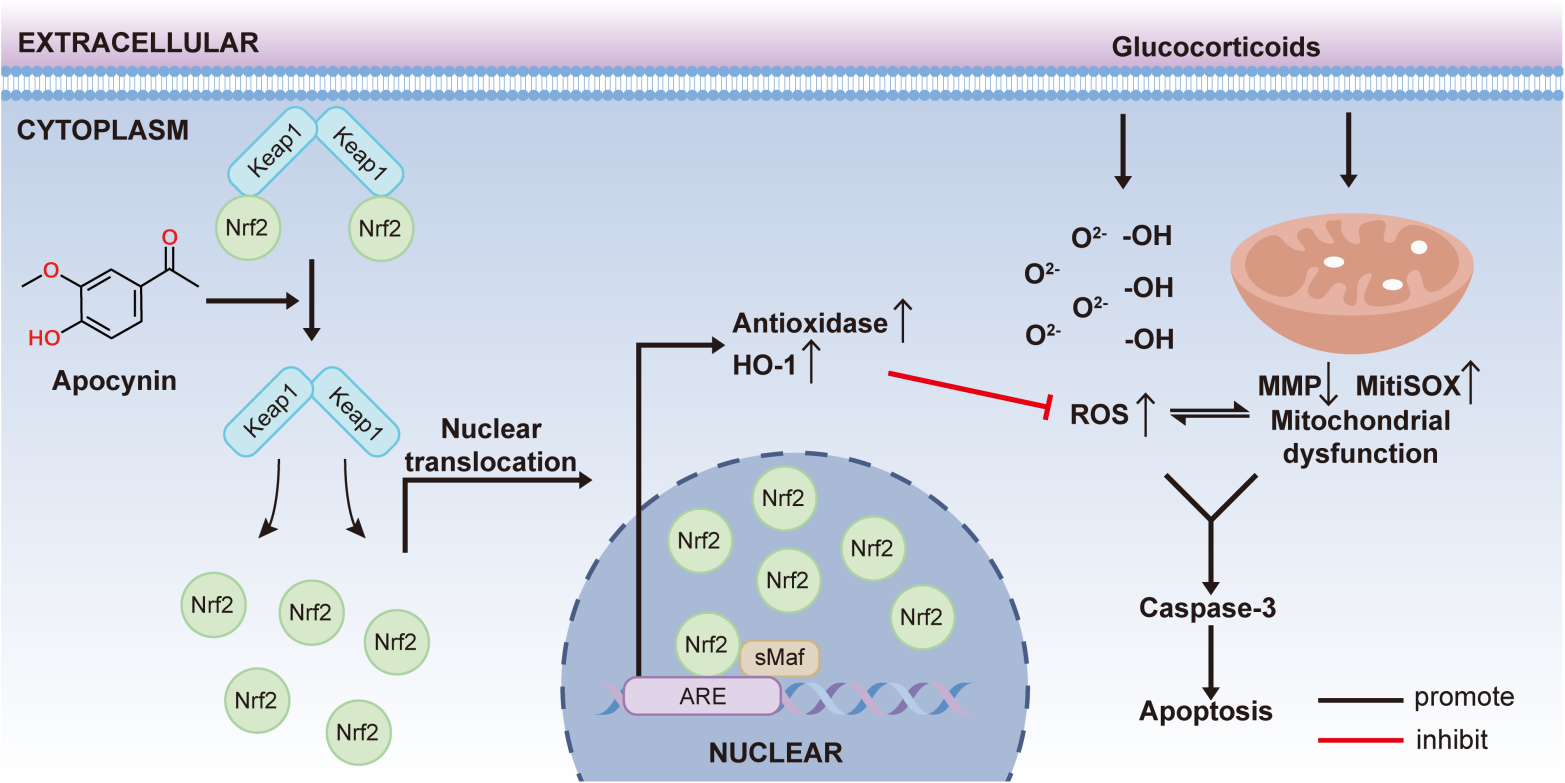
Schematic model of the osteoprotective effect of APO. APO promotes the activation of Nrf2 pathway to reduce cellular ROS and restore the MMP.

We investigated the effect of APO treatment on the expression of Nrf2, HO‐1 and antioxidant enzymes in a rat model of SIFHN. As shown in Figure 9A,B, compared with those in the Dex group, APO pretreatment significantly elevated the levels of Nrf2 and HO‐1 and decreased the levels of GSH, CAT, GPx and SOD (Figure 9E–H) in rat serum in the Dex + APO group. APO also decreased the levels of the lipid peroxidation products 4‐HNE (Figure 9C) and 8‐OhdG (Figure 9D). Our findings indicated that APO inhibited Dex‐induced OS by activating Nrf2 to promote the expression of downstream antioxidant enzymes, thereby alleviating the progression of SIFHN (Figure 10).

## 4 DISCUSSION

The incidence of SIFHN is increasing year by year and accounts for the largest proportion of non‐traumatic femoral head necrosis. Cumulative or high‐dose shock therapy with GCs is the most important risk factor for SIFHN.^26^ Since 2020, when COVID‐19 spread worldwide, patients who were severely infected with coronaviruses have received high‐dose, pulsed treatment with GCs, which may cause these patients to progressively develop SIFHN during the following years. However, 67 per cent of patients with SIFHN are asymptomatic in the early stages,^27^ and Shimuzu et al.^28^ reported that the progression from ARCO Stage I to Stage IV takes an average of 49 months. Therefore, to effectively treat SIFHN, early and prompt intervention, the identification of prospective targets and the development of effective medications are essential and urgent. In the current work, we assessed the effect of APO on Dex‐mediated osteoblast apoptosis. Our findings suggested that APO protected osteoblasts from Dex‐mediated OS and mitochondrial functional impairment. Mechanistically, APO suppressed intracellular ROS generation and restored mitochondrial function through activation of the Nrf2 pathway. In vitro, APO inhibited apoptosis and attenuated osteonecrosis by increasing the expression of Nrf2 in the rat femoral head.

APO is a naturally occurring acetophenone found in rooibos and black walnut roots. Initially, APO was primarily reported to be a NOX inhibitor. However, it has now been demonstrated that APO can alleviate a wide variety of conditions, including diabetes‐related complications, neurodegenerative disorders, cardiovascular diseases, cancer and ischaemia–reperfusion injury.^17^ Stéphane M. Camus et al.^29^ proved that APO reduced ROS generation and inhibited endothelial cell apoptosis in sickle cell disease. In addition, APO protected osteoblasts from antimycin A‐induced mitochondrial dysfunction and apoptosis.^30^ Targeted delivery of mitoapocynin effectively alleviated ROS production in mitochondria, showing promising therapeutic potential.^31^ Consistent with previous findings, we showed that APO promoted cell survival and reversed changes in C‐caspase3, BAX and BCL‐2 expression in Dex‐induced osteoblasts.

OS and mitochondrial dysfunction‐mediated apoptosis in osteoblasts play a pivotal role in GC‐induced bone metabolism abnormalities.^5^ Numerous reports have shown that GCs promote the accumulation of ROS in areas of osteonecrosis by inducing vascular dysfunction, intraventricular hypertension, osteoblast apoptosis, inflammatory factor production and other related factors that contribute to tissue ischaemia and hypoxia.^24^, ^32^, ^33^, ^34^ Excessive ROS attack macromolecules, including proteins, DNA and lipids, and trigger pathological mitochondrial clearance and cell apoptosis via ROS‐induced ROS release.^10^, ^35^ GCs influence mitochondria by modifying the expression of mitochondrial and nuclear genes and reducing the MMP by binding to their receptors on the mitochondrial membrane.^36^, ^37^ The loss of MMP, which is an early pathologic event in apoptosis, promotes the release of intracellular cytochrome C, which activates C‐caspase3 and ultimately triggers the intrinsic mitochondrial apoptotic pathway.^35^ Our results suggested Dex markedly raised the levels of ROS and aggravated OS injury, thus disrupting the MMP. These changes lead to apoptosis. Encouragingly, APO significantly reduced ROS levels in cells, restored mitochondrial function and inhibited the activation of C‐caspase3.

Weakened mechanical strength in bone trabeculae and reduced osteogenic differentiation in the subchondral bone region of the femoral head are important factors in the development of collapse during SIFHN.^5^, ^38^ Eric E. Beier et al.^39^ demonstrated that GCs significantly accelerated bone loss and prevented osteoblasts from forming mineralized nodules, which resulted in microfractures of the trabeculae and a decline in trabeculae‐related parameters, such as trabecular volume (TV), Tb.N and Tb.Th, as well as a corresponding decline in BMD. However, APO rescued the deterioration of mechanical strength and the mineralization properties of bone trabeculae in the subchondral bone region. This may be another crucial function of APO in delaying the collapse and progression of SIFHN.

The Nrf2 pathway plays a crucial antioxidant role in cells.^40^ Nrf2 translocates from the cytoplasm into the nucleus and promotes the transcription of downstream antioxidant enzyme.^41^, ^42^ Geng et al.^43^ showed that GCs suppress the Nrf2 signalling pathway and the antioxidant capacity of tissues. GSH bind with lipid peroxide and free radicals to resist the destruction of sulfhydryl groups by oxidants.^44^ In our study, APO promoted the expression of Nrf2 and HO‐1. In addition, APO enhanced antioxidant enzyme activity in cells and animal serum. These results suggested that APO attenuated oxidative stress‐induced damage in Dex‐induced osteoblasts through Nrf2 pathway activation.

In the present study, APO treatment inhibited osteoblast apoptosis induced by OS and mitochondrial dysfunction via the Nrf2 signalling pathway. However, the in‐depth mechanism needs to be further explored. The potential mechanism of Nrf2 activation by APO may be as follows: (1) APO treatment may weaken the binding ability of GCs to GC receptors (GRs) and inhibit downstream gene transcription. (2) APO may attenuate GR activity. (3) In the physiological state, the binding of Keap1 and Nrf2 is the main mediator of Nrf2 ubiquitination and degradation. APO inhibits the degradation of Nrf2 by facilitating the dissociation of KEAP1 and Nrf2.^45^ (4) The interaction between Keap1 and Nrf2 can be altered by the phosphorylation of Nrf2, and APO may regulate Nrf2 phosphorylation through protein kinases. In addition, studies have revealed that osteoclast overactivation plays a critical role in the progression of SIFHN, while APO may alleviate SIFHN by inhibiting osteoclastogenesis.^46^

## 5 CONCLUSION

In summary, APO inhibits apoptosis in Dex‐treated osteoblasts. APO protects osteoblasts from Dex‐mediated oxidative stress and mitochondrial impairment by activating Nrf2 signalling. In addition, APO mitigates the occurrence and progression of SIFHN in rats. These results provide theoretical support for the pharmacological treatment of SIFHN. APO may be a promising targeted medicine for the management of steroid‐induced necrosis of the femoral head.

## AUTHOR CONTRIBUTIONS

**Xinglong Zhang:** Conceptualization (equal); methodology (equal); writing – original draft (equal). **Ran Pang:** Methodology (supporting). **Kai Zhang:** Methodology (supporting); writing – original draft (supporting). **Qian Xu:** Writing – review and editing (equal). **Chunlei Xu:** Methodology (supporting). **Wei Shi:** Data curation (equal). **Xinyu Liang:** Methodology (equal). **Dong Li:** Data curation (equal). **Wenhao Cui:** Writing – review and editing (equal). **Shucai Bai:** Data curation (equal). **Zhijun Li:** Writing – review and editing (equal). **Hui Li:** Conceptualization (equal); project administration (equal). **Huafeng Zhang:** Conceptualization (lead); project administration (lead). **Xinglong Zhang**, **Ran Pang**, and Kai Zhang contributed equally to this work and should be considered co‐first authors.

## FUNDING INFORMATION

There is no funding to report.

## CONFLICT OF INTEREST STATEMENT

There are no conflicts of interest to declare.

## Data Availability

All original data in this article can be obtained from the corresponding author upon reasonable request.
